# Rosaceae Honey: Antimicrobial Activity and Prebiotic Properties

**DOI:** 10.3390/antibiotics14030298

**Published:** 2025-03-13

**Authors:** Francesca Coppola, Manar Abdalrazeq, Florinda Fratianni, Maria Neve Ombra, Bruno Testa, Gokhan Zengin, Jesus Fernando Ayala Zavala, Filomena Nazzaro

**Affiliations:** 1 Institute of Food Science, CNR, Via Roma 64, 83100 Avellino, Italy; francesca.coppola2@unina.it (F.C.); fratianni@isa.cnr.it (F.F.); nives.ombra@isa.cnr.it (M.N.O.); 2 Department of Food Science, University Federico II, Via Università 100, Portici, 80055 Naples, Italy; 3 Department of Biomedical Sciences, Faculty of Medicine and Health Sciences, An-Najah National University, Nablus 00970, Palestine; manar.abdalrazeq@najah.edu; 4 Q Center, Biomedical Department, Global University College of Science and Health (GUCSH), Rawabi, Palestine; 5 Department of Agricultural, Environmental and Food Sciences, University of Molise, Via De Sanctis, 86100 Campobasso, Italy; bruno.testa@unimol.it; 6 Department of Biology, Faculty of Science, Selcuk University, 42250 Konya, Turkey; gokhanzengin@selcuk.edu.tr; 7 Centro de Investigación en Alimentación y Desarrollo, A.C. (CIAD), Carretera Gustavo Enrique Astiazarán Rosas, No. 46, Col. La Victoria, Hermosillo 83304, Sonora, Mexico; jayala@ciad.mx

**Keywords:** biofilm, honey, probiotic, prebiotics

## Abstract

**Background:** Flowering members of the globally diffused Rosaceae family include popular plants, such as apple, almond, and cherry, which play a fundamental role as honeybee nectariferous and polleniferous agents. Through the production of honey, these plants can also play an indirect role in the prevention and treatment of many diseases, including infections, fighting the occurrence of resistant microorganisms, and concurrently stimulating the growth of beneficial bacteria. **Objectives:** This study focused on the effect of some Rosaceae plants’ honey, including hawthorn, cherry, raspberry, almond, and apple, against the pathogens *Acinetobacter baumannii*, *Escherichia coli*, *Klebsiella pneumoniae*, *Listeria monocytogenes*, *Pseudomonas aeruginosa*, and *Staphylococcus aureus*. **Results:** Results demonstrated the honey’s ability to impair swimming motility. A crystal violet test indicated that honey could inhibit the formation and stabilization of biofilms, with inhibition rates up to 59.43% for immature biofilms (showed by apple honey against *A. baumannii*) and 39.95% for sessile bacterial cells in mature biofilms (when we used cherry honey against *S. aureus*). In the test with 3-(4,5-dimethylthiazol-2-yl)-2,5-diphenyltetrazolium bromide, cherry and apple honey were the most effective in inhibiting sessile cell metabolism honey in both immature (56.47% cherry honey vs. *K. pneumoniae*) and mature biofilms (54.36% apple honey vs. *A. baumannii*). Honey stimulated the growth of *Lactobacillus bulgaricus*, *Lacticaseibacillus casei Shirota*, *Lactobacillus gasseri*, *Lacticaseibacillus plantarum*, and *Lacticaseibacillus rhamnosus*; hawthorn, raspberry, and almond honey significantly increased the in vitro adhesion capacity of *L. bulgaricus* and *L. casei* Shirota. Tests with probiotic supernatants demonstrated honey’s ability to inhibit the biofilm formation and metabolism of the pathogens. **Conclusions**: Our results encourage further studies to assess the potential application of Rosaceae honey for food preservation and in the health field, as it could fight the antimicrobial resistance of food and clinical pathogens, and potentially enhance the host’s gut wellness. The use of honey for nanotechnological and biotechnological approaches could be suggested too.

## 1. Introduction

Antimicrobial resistance is a growing concern and a significant challenge for healthcare systems worldwide. In regions such as the EU, approximately 70% of the total burden comes from the three most critical antibiotic-resistant pathogens. According to WHO reports (2022), infectious diseases are the second leading cause of death globally, resulting in around 13 million fatalities each year [1]. Furthermore, in 2024, the World Economic Forum’s Global Risks Report identified antimicrobial resistance as one of the most serious threats to human health [2]. Multidrug-resistant bacterial infections account for 25,000 deaths annually in Europe. This draws attention to the urgency of the issue within the healthcare sector, making it a primary concern rather than a secondary one. In recent years, some bacteria, such as *Staphylococcus aureus*, *Klebsiella pneumoniae*, *Acinetobacter baumannii*, *Pseudomonas aeruginosa*, and *Enterobacter* species (ESKAPE) family organisms, have become emerging challenges as multidrug-resistant organisms [3,4] characterized by the capacity to form biofilms, self-produced extracellular matrixes composed of polysaccharides, proteins, and nucleic acids, on many surfaces in natural and clinical environments, leading to significant food, ecological, and medical challenges. Sessile bacterial cells within biofilms change their metabolic pathways and often exhibit increased antibiotic resistance, ascribable to physical, biochemical, structural, and genetic factors [5]. Consequently, infections characterized by the formation of biofilms are often more persistent and difficult to eradicate, leading to more significant morbidity. The formation of biofilms can lead to various infectious diseases. As reported by Zafer et al. (2024), according to the National Institute of Health, bacterial biofilms are responsible for 65% of microbial infections and 80% of chronic infections [6]. As resistance rises, treating infections under these complex conditions is becoming increasingly challenging [7]. These issues highlight the critical role of both antimicrobial resistance and biofilms in infection management, emphasizing the need for focused attention and intervention. Conventional treatments frequently fall short in their effectiveness against infections associated with biofilm, which causes the exploration of alternative strategies. Exploring other natural agents offers an intriguing route for possible antibiotic activity. Multiple research efforts are presently investigating natural and non-antibiotic substances as potential substitutes or supplementary therapies. These include also honey and microbial-based treatments such as prebiotics, probiotics, synbiotics, and postbiotics, among others [8,9].

Exploring honey’s role as an antibiofilm agent is particularly intriguing since it offers advantages over traditional treatments and aligns with the growing emphasis on natural and holistic approaches to health and environmental management. Compared with conventional antibiotics, the potential multitarget antimicrobial activity of honey is often linked to a reduced risk of developing resistance in microorganisms. Furthermore, honey significantly promotes probiotic bacterial growth and functionality [10,11,12]. Thanks to its prebiotic compounds, honey promotes the growth and activity of beneficial bacteria like lactobacilli and bifidobacteria. Honey is recognized as a functional food and can be found in various functional products, cosmetics, and other applications, providing multiple health benefits. Consumers’ growing interest in honey with specific tastes, smells, and potential health advantages drives demand for monofloral honey. Monofloral honey, obtained predominantly from the nectar of individual blooming plants, provides consumers with a more tailored and premium honey experience, frequently connected with specific geographical terroirs and unique floral flavors. The interest in organic honey is due to the rising consumer interest in its production through organic beekeeping that respects the environment and the normal life cycle of bees and to a huge demand for natural and healthy sweeteners for culinary applications linked to health advantages. Acacia, sider, clover, orange, ajwain, manuka, and buckwheat are the most marketed monofloral honey varieties [13]. Honey’s antimicrobial properties operate through various mechanisms. It can alter bacterial traits, such hydrophobicity, reducing their ability to adhere to human cells. Additionally, honey can inhibit or diminish pathogens’ capacity to form biofilms, inducing changes in bacterial sessile cells and reducing their virulence [14,15,16,17,18,19,20]. Thus, honey exhibits a broad spectrum of microbiological benefits.

Plants are known for their potent biological activities, which offer numerous health benefits [21]. The Rosaceae family is a particularly significant group, encompassing approximately 5000 species across 110 genera. These species hold immense economic value, serving diverse roles as food sources, ornamental plants, medicinal resources, forage, and raw materials for industrial use.

Extracts obtained from parts of plants or Rosaceae waste showed antimicrobial and antibiofilm activity mainly against *Acinetobacter baumannii*, *Escherichia coli*, *Pseudomonas aeruginosa*, *Staphylococcus aureus*, and *Klebsiella pneumoniae* [22,23,24,25].

Considering the interest sparked by plants in the Rosaceae family, which includes several nectariferous species, and the well-established significance of monofloral honeys in the nutraceutical field, as highlighted by previous studies, we aimed to assess the potential antimicrobial activity of certain types of Rosaceae honey against pathogens, and to explore their prebiotic effects on specific lactic acid bacteria, of technological and health-related interest [26,27].

## 2. Results

Our study pursued distinct investigative approaches: on the one hand, we analyzed the antimicrobial activity of Rosaceae honey by evaluating its effect on some characteristics of some pathogenic strains, such as swimming, biofilm formation and reinforcement, and the metabolic activity of their sessile cells. Simultaneously, we examined the honey’s prebiotic effects on some commercially available probiotic strains. For this, we assessed how replacing glucose with honey in standard MRS growth medium influenced probiotic growth and hydrophobicity, a key factor in their potential ability to adhere to the intestinal epithelium. In addition, we utilized the supernatants from probiotic cultures to further explore their postbiotic effects by evaluating antibiofilm activity against the abovementioned pathogens.

### 2.1. Antibiofilm Activity of Honey

In our study, following the evaluation of the minimal inhibitory activity (Table 1), we utilized the crystal violet (CV) assay to assess the ability of Rosaceae honey to interfere with the adhesion phase of the six pathogens and to impact their mature biofilms.

Additionally, we applied the MTT assay to determine whether the presence of honey in the culture medium could suppress the metabolic activity of sessile bacterial cells. The results of the CV and MTT tests are shown in Table 2 and Table 3, respectively.

Adding honey at the beginning of bacterial incubation considerably reduced the adhesion process in most cases. *Acinetobacter baumannii*, *Escherichia coli*, *Listeria monocytogenes*, and *Staphylococcus aureus* were the most affected bacterial strains, with inhibition rates ranging from 34.30% (raspberry honey against *S. aureus*) to 59.43% (apple honey against *A. baumannii*).

In the test with crystal violet, honey, added at the beginning of the growth of *A. baumannii*, a Gram-negative bacterium well known for its ability to resist multiple antibiotic treatments [28,29,30], determined a potent inhibition of biofilm that was never below 43.49%, which was also observed with hawthorn honey, compared to the control. For *E. coli*, for which the biofilm is the principal causative agent for recurrent urinary tract infections and indwelling medical device-related infectivity [31], the inhibitory effect of the honey was up 57.59% and consistently above 47.42%, with the lowest inhibition observed using hawthorn honey. In the case of *L. monocytogenes*—recognized as a problem for the food industry due to its environmental persistence, also attributed to its ability to form biofilms [32]—the honey maintained an inhibitory effect up to 55.87% and was consistently above 44.53%, with this minimum recorded when apple honey was added at the start of bacterial growth. The immature biofilm of *S. aureus* was inhibited mainly by the presence of cherry honey (51.25%); on the other hand, the lowest inhibition (28.76%) was observed when the microorganism was incubated with almond honey.

*Pseudomonas aeruginosa* and *Klebsiella pneumoniae* displayed greater resistance to the honey, varying their effects by strain. For instance, apple and raspberry honey did not act against *K. pneumoniae* but demonstrated moderate inhibitory capability (22.73% and 31.88%, respectively) vs. *P. aeruginosa*. Conversely, cherry honey, without impacting *P. aeruginosa* biofilm formation, exhibited an inhibitory effect of 29.66% when added at the beginning of *K. pneumoniae* growth. Finally, almond and hawthorn honey showed minimal or no inhibitory effects on their biofilm formation.

The inhibitory effects of Rosaceae kinds of honey varied when we tested them against the mature biofilms of the pathogenic strains. In most cases, their presence was either ineffective or minimally effective, with a few notable exceptions. For example, in the case of *P. aeruginosa*, cherry honey, which had no impact on biofilm formation, demonstrated an inhibitory effect of 20.23% against the mature biofilm of this strain. Similarly, almond honey, whose action was weak against the immature biofilm, showed an inhibitory effect of 33.08% when applied to the mature biofilm of that species. Interestingly, apple honey was more effective against the mature biofilm of *P. aeruginosa* (38.01%) than it was against its immature biofilm.

All kinds of honey, except hawthorn, retained some inhibitory activity against the mature biofilm of *Staphylococcus aureus* (with inhibition percentages ranging from 10.95% for almond honey to 39.95% for cherry honey) and *A. baumannii* (with inhibition percentages ranging from 2.29 to 14.33%).

The MTT assay enabled us to evaluate how Rosaceae honey’s presence influenced sessile cells’ metabolic activity within microbial biofilms. The results are summarized in Table 3. When honey was added at the beginning of the *A. baumannii* incubation, it had little effect on its metabolic activity. Only cherry honey (27.16% inhibition) and, to a lesser extent, apple honey (2.57% inhibition) demonstrated any influence. The inhibitory effects of the other kinds of honey appear to stem from currently unidentified mechanisms. Conversely, the Rosaceae honey had a more pronounced impact on the metabolism of sessile cells within the mature biofilm of *A. baumannii*. All kinds of honey, except cherry honey, which showed no metabolic inhibition, could reduce bacterial metabolism, with inhibition percentages reaching 54.36% (apple honey) and no lower than 32.2% (raspberry honey).

For *E. coli*, MTT results indicated that all five Rosaceae types of honey inhibited the metabolism of sessile cells within the immature biofilm. However, they did not show an inhibitory effect on the metabolism of sessile cells in the mature biofilm, except for apple honey, which, while not impacting the biofilm itself, did influence its metabolism (41.54% of inhibition).

In the case of *K. pneumoniae*, honey did not affect biofilm formation but inhibited the metabolism of its sessile cells in the immature biofilm. The inhibition ranged from 24.24% (apple honey) to 56.47% (cherry honey). On the contrary, honey did not affect the metabolism of sessile cells within the mature biofilm.

Regarding *L. monocytogenes*, Rosaceae honey had a marked inhibitory effect on both the immature biofilm and the metabolic activity of its sessile cells, with similar percentages. However, this effect was not observed in mature biofilm. In that scenario, metabolic inhibition was minimal, with percentages of 5.36%, 5.49%, and 1.7% for cherry, apple, and almond honey, respectively.

The five types of Rosaceae honey effectively inhibited the metabolism of sessile cells within the immature biofilm of *P. aeruginosa*, except almond and apple honey. Interestingly, although cherry honey was ineffective in other aspects, it exhibited the highest inhibitory effect on the metabolism of sessile cells for this strain (25.66%). This suggests that cherry honey may still counteract the pathogenicity of *P. aeruginosa*, potentially targeting specific mechanisms involved in metabolic changes that enhance its virulence.

Only raspberry honey showed inhibitory activity, albeit modestly, on the mature biofilm of *P. aeruginosa*, with a metabolic inhibition of 15.91%.

The sessile cells of *S. aureus* immature biofilm were highly resistant to the honey’s metabolic effects. However, the sessile cells present within the mature biofilm showed some sensitivity, particularly to almond honey (5.17% inhibition) and, more notably, to apple honey, which achieved an inhibition of 19.52%.

### 2.2. Swimming Motility

The pathogens’ exposure to the five kinds of Rosaceae honey reduced swimming motility in most cases. Results are shown in Table 4.

*L. monocytogenes* was the most susceptible bacteria to the effects of all five types of honey, which decreased its motility by 17% to 42%. This is an auspicious result. *L. monocytogenes*, through motility, can increase the aggressive action of epithelial invasion during intestinal infection [33]. *A. baumannii* and *S. aureus* exhibited slightly higher resistance, with *A. baumannii* showing no response to almond and apple honey, and *S. aureus* being unaffected by hawthorn and cherry honey. Notably, cherry honey significantly inhibited pathogen motility, with reductions ranging from 33.33% to 52.33% (against *E. coli*), highlighting its potential as a powerful natural treatment.

### 2.3. The Prebiotic Activity of Rosaceae Honey

Our study investigated the influence of honey on commercially available probiotic strains (*L. casei*, *L. gasseri* Shirota, *L. plantarum*, and *L. rhamnosus*) and a collection strain, *L. bulgaricus*, whose health benefits have been highlighted in the literature [34]. Specifically, we evaluated honey’s effects on these microorganisms’ growth, proliferation, and hydrophobicity levels. Additionally, we assessed whether the growth supernatant media had antibiofilm activity against the six pathogenic strains indicated above and whether such activity differed from that observed in supernatants from microorganisms grown in standard MRS medium (containing glucose). The results are presented in Table 5, Table 6, Table 7 and Table 8. The behavior of five lactic acid bacteria when grown in the presence of Rosaceae honey as a replacement for glucose in the MRS formula is shown in Table 5. The data are related to the spectrophotometric measurements performed at a wavelength of λ = 600 nm.

All types of Rosaceae honey promoted the growth of lactic acid bacteria. This growth-promoting effect was particularly pronounced for *L. plantarum* and *L. rhamnosus*, whose growth increased nine to ten times compared to their respective controls. Specifically, starting from an initial optical density of 0.125 and 0.182, the addition of honey resulted in growth that achieved λ values no lower than 1.331 and 1.376, respectively, when cultured in the presence of cherry honey. For the other three probiotic strains, *L. bulgaricus* grown in conventional MRS medium gave a λ value = 0.603. However, the presence of honey consistently doubled this value. Cherry honey, while slightly less effective than other types of honey, still demonstrated a significant growth-enhancing effect. Similarly, *L. casei* Shirota growth with honey exhibited nearly a threefold increase, reaching λ values of 1.512 and 1.622 in the presence of hawthorn and raspberry honey, respectively. Even with cherry honey, which was marginally less effective, bacterial growth reached a λ value of 1.322. A comparable pattern was observed for *L. gasseri*, where the λ value increased from 0.449 in the control to 1.431 when the strain was grown with raspberry honey. This highlights the consistent ability of different Rosaceae honeys to enhance the growth of these probiotic bacteria.

#### 2.3.1. Microbial Adhesion to Solvent

The evaluation of cell surface hydrophobicity indicates a probiotic’s ability to adhere to intestinal epithelial cells [35,36]. The affinity for hydrocarbons, such as xylene, can be considered a biochemical marker for adhesion to gut epithelial cells [37]. The impact of honey on probiotic bacterial strains’ growth and in vitro adhesion capacity was evaluated by comparing their growth under standard conditions (in MRS broth). Specifically, we analyzed the cell surface hydrophobicity of *L. bulgaricus*, *L. gasseri*, *L. casei Shirota*, *L. plantarum*, and *L. rhamnosus*. Results are shown in Table 6.

The behavior of the bacteria varied depending on the type of honey used in the test. For *L. bulgaricus*, the presence of honey consistently increased hydrophobicity regardless of honey type. Starting from a baseline hydrophobicity value of 3.71% in the control, the value rose to 4.66% with cherry honey and reached 10.50% and 12.70% when hawthorn and almond honey were used as fermentable substrates, respectively. Also, with apple and raspberry honey, the hydrophobicity values were significantly elevated, doubling to 7.97% and nearly tripling to 8.95%.

In contrast, *L. casei Shirota*, which exhibited a very low control hydrophobicity value (0.50%), displayed significantly higher values when grown with almond honey (2.26%), hawthorn honey (4.23%), and especially raspberry honey, which yielded a hydrophobicity value of 10.13%. For *L. gasseri*, honey-induced hydrophobicity increased only with apple honey (4.07%) and raspberry honey (6.57%). Meanwhile, *L. plantarum* showed a notable increase in hydrophobicity with cherry honey, rising from 0.78% in the control to 5.87%, an eightfold increase.

#### 2.3.2. Antibiofilm Activity Exhibited by the Probiotics’ Culture Supernatants

##### Crystal Violet Assay

Hawthorn honey, in particular, frequently enhanced the antibiofilm capacity of the *L. bulgaricus* supernatant. Notably, in the case of *L. bulgaricus*, the inhibition rate against *K. pneumoniae* increased more than double compared to the control (72.15% vs. 33.06%), as well as against *L. monocytogenes* (44.35% compared to 17.69% for the control). Additionally, the supernatants of *L. bulgaricus* grown with hawthorn and raspberry honey showed inhibitory effects against *P. aeruginosa*, which exhibited complete resistance to the supernatant of the probiotic grown in glucose. In contrast, cherry honey was largely ineffective in enhancing the antibiofilm capacity of the culture supernatant, except for modest inhibitory effects observed against *A. baumanni* (49.33%) and *S. aureus* (18.07%). For *L. casei* Shirota, Rosaceae honey used as a fermentable substrate instead of glucose had varying effects on the antibiofilm activity of the culture supernatants, depending on the type of honey and the pathogen tested in the CV assay. Specifically, almond honey (inhibition = 57.99%) and apple honey (inhibition = 53.71%) enhanced the antibiofilm activity of the supernatant against *A. baumannii* compared to the control supernatant (inhibition = 43.01%). The presence of honey in the culture broth of *L. casei* Shirota generally had no effect against *E. coli*, except for apple honey, which increased the inhibition from 18.96% (control) to 26.35%. Hawthorn honey had the most significant impact against *K. pneumoniae*, doubling the inhibitory efficacy of the supernatant from 34.22% (control) to 72.15%. However, the supernatants’ antibiofilm activity against *L. monocytogenes* was unaffected by substituting glucose with honey, as the control supernatant containing glucose consistently showed the highest inhibitory efficacy (63.95%). Similarly, no significant improvement in inhibitory activity was observed for the *L. casei* Shirota culture supernatants when tested against *P. aeruginosa* (except for hawthorn honey) and *S. aureus*.

The *L. gasseri* supernatant was generally ineffective in the crystal violet assay against the six pathogens. Notable exceptions included its antibiofilm activity against *P. aeruginosa* and *S. aureus* when grown in the presence of cherry honey, achieving inhibition rates of 34.43% and 60.62%, respectively. Additionally, the *L. gasseri* supernatants grown with raspberry honey (28.02%) and apple honey (27.72%) showed antibiofilm activity against *S. aureus*.

The supernatant of *L. plantarum* demonstrated notable antibiofilm activity against *A. baumannii* when the probiotic strain was cultivated with honey. Inhibition values ranged from 13.27% (observed with cherry honey) to 50.71% (achieved with hawthorn honey). In other cases, honey either failed to enhance the antibiofilm efficacy or even eliminated it. As for *L. rhamnosus*, its growth in MRS alone showed minimal antibiofilm activity in the culture supernatant. However, using hawthorn honey as a fermentable substrate improved its antibiofilm effectiveness, particularly against *S. aureus* (16.99%) and even more so against *P. aeruginosa*, with an observed inhibition of 44.16%.

##### 3-(4,5-Dimethylthiazol-2-yl)-2,5-Diphenyltetrazolium Bromide (MTT) Test

The MTT assay demonstrated that the biofilm inhibitory activity might stem from effects on the metabolism of sessile cells in the pathogens (Table 8). This was particularly evident for *E. coli*, whose metabolism was almost entirely suppressed by the culture supernatant of *L. bulgaricus* grown with hawthorn honey. Significant inhibitory effects were also observed against *A. baumannii* when the *L. bulgaricus* supernatant was derived from cultures with almond (67.64%) or apple (62.81%) honey. Similarly, against *S. aureus*, the supernatant of *L. bulgaricus* grown with all honey types, except hawthorn honey, exhibited strong inhibitory activity. The inhibitory action was especially notable when honey was included in the culture medium of *L. casei* Shirota. The resulting supernatants effectively inhibited the metabolism of sessile cells for *A. baumannii*, *E. coli*, and *K. pneumoniae*. However, their effectiveness was less pronounced against *L. monocytogenes*, *P. aeruginosa*, and *S. aureus*. The *L. gasseri* supernatant consistently inhibited the metabolism of sessile cells for *A. baumannii*, *K. pneumoniae*, and *E. coli*. However, the presence of honey as a fermentable substrate only occasionally enhanced its efficacy compared to the control, which itself recorded inhibition values exceeding 89% (e.g., against *K. pneumoniae*). Adding honey to the culture medium did not alter the outcome for *L. monocytogenes* and *P. aeruginosa*, where the control supernatant was largely ineffective. Adding honey diminished the inhibitory effect on sessile cell metabolism against *S. aureus*. Supernatants of the cultures of *L. plantarum* grown with honey displayed notable inhibitory effects, reaching up to 58.47% against *A. baumannii* (with raspberry honey). This contrasted with the control, which did not show inhibition. Similarly, almond honey enhanced the inhibitory effects of *L. plantarum* supernatant against *E. coli* and *K. pneumoniae*. All tested kinds of honey improved the efficacy of *L. plantarum* supernatants against *S. aureus*. However, honey generally did not significantly increase the inhibitory efficacy of *L. rhamnosus* supernatants compared to the control, which already demonstrated potent metabolic inhibition across all six pathogens. The inhibitory efficacy exceeded the controls in only a few cases. For instance, with *L. rhamnosus*, honey enhanced its inhibitory effectiveness against *A. baumannii* (cherry honey, raspberry honey, almond honey, and apple honey) and *P. aeruginosa* (hawthorn honey).

**Figure 1:**
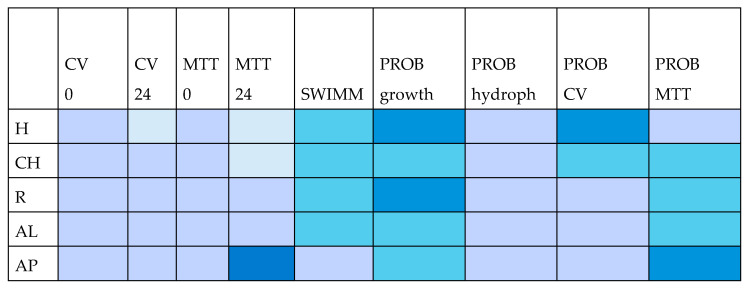
A visual representation showing the antimicrobial and prebiotic effectiveness of hawthorn (H), cherry (CH), raspberry (R), almond (AL), and apple (AP) honey. CV: crystal violet test; MTT: MTT test; SWIMM: evaluation of swimming motility of pathogenic bacteria; PROB probiotic. The color intensity corresponds to the honey’s performance, reflecting its higher or lower effectiveness (the more intense the color, the more performant the honey) based on the tests’ outcomes.

Figure 1 visually summarizes the antimicrobial and prebiotic effectiveness of the five kinds of Rosaceae honey.

All types of honey exhibited a strong capacity to inhibit biofilm formation and the metabolism of pathogenic sessile cells when introduced during the early stages of bacterial growth. Raspberry, cherry, apple, and almond honey showed notable activity against mature pathogenic biofilms, which are intricate structures that are complicated to target. Apple honey was the only one capable of inhibiting the mature biofilm of *K. pneumoniae*, although it did not affect its metabolism, so it could be valuable as food support for vulnerable groups, such as infants and the elderly, who are more frequently subjected to pulmonary infection. Furthermore, while it had no impact on the mature biofilm of *E. coli*, it was the only one to inhibit the metabolism of its sessile cells in both the immature and mature biofilms. This highly irreversible condition generally leads to increased bacterial pathogenicity traits and, among other things, to greater antibiotic resistance. When tested against the metabolism of *A. baumannii* mature biofilm, it even improved its performance since, despite being completely ineffective on the metabolism of cells embedded in the immature biofilm, it showed an even better inhibitory action, as evidenced by the inhibition percentage value. Additionally, all kinds of honey—particularly hawthorn, cherry, raspberry, and almond—significantly reduced the motility of pathogenic bacteria.

The prebiotic activity of Rosaceae honey was particularly evident when honey replaced glucose in the broth culture, as compared to the control, which used standard MRS broth. The supernatants from probiotic cultures grown with honey, especially hawthorn and cherry honey, showed strong biofilm-inhibitory properties. In general, all types of Rosaceae honey, except for hawthorn, effectively reduced the metabolism of pathogenic sessile cells. Once again, apple honey demonstrated particularly high activity.

## 3. Discussion

Research on the antimicrobial activity of Rosaceae honey is limited, and studies focusing on their antibiofilm properties are even rarer. Some findings revealed a particular activity of some types of Rosaceae honey, such as the old wild cherry honey studied by Pătruică et al. (2021), against *E. coli*, *Candida albicans*, and *Bacillus cereus*, with inhibition rates reaching up to 47.35%. The authors also observed that wild cherry honey was ineffective against *Shigella flexneri*, *P. aeruginosa*, and *L. monocytogenes*. On the other hand, the activity was very weak against *Streptococcus pyogenes*, *S. aureus*, *Salmonella typhimurium*, and *Candida parapsilopsis* [38]. Except for this work, we did not find other studies on the antimicrobial activity of cherry. Similarly, blackberry honey demonstrated antimicrobial activity, particularly against *S. aureus* and *L. monocytogenes* [39]. Honey of genus *Rubus*, analyzed by Escuredo et al. (2012), showed antibacterial capacity against *Staphylococcus* (*S. aureus*, *S. epidermidis*), *Proteus*, *E. coli*, *Micrococcus luteus*, and *S. typhimurium* [40]. Recently, Saftic Martinovic et al. (2024) evaluated the antimicrobial activity of raspberry honey, which exhibited moderate antimicrobial activity against *S. aureus* species [41]. These findings underline the potential of Rosaceae honey in combating specific pathogens while highlighting the variability in their efficacy depending on the bacterial strain.

### 3.1. Antibiofilm Activity

Our results differed from those reported by Nazzaro et al., who analyzed the effects of various types of Lamiaceae honey added at the onset of *A. baumannii*, *E. coli*, *L. monocytogenes*, *P. aeruginosa*, and *S. aureus* growth [19]. Their study highlighted that honey inhibited bacterial adhesion and subsequent biofilm formation, particularly against *A. baumannii* and, even more so, against *S. aureus*. However, the authors used different honey concentrations in their experiments. Our results reveal that these kinds of honeys demonstrated noticeable biofilm inhibitory activity in some cases.

Apple honey was the only one capable of inhibiting the mature biofilm of *K. pneumoniae*, although it did not affect its metabolism. However, while it had no impact on the mature biofilm of *E. coli*, it was the only one to inhibit the metabolism of its sessile cells in both the immature and mature biofilm. This last condition, which is highly irreversible, generally leads to increased bacterial pathogenicity traits and, among other things, to greater antibiotic resistance. It improved its performance since, despite being completely ineffective on the metabolism of cells embedded in the mature biofilm, it was capable of acting on the mature biofilm of *A. baumannii*, achieving an even higher inhibitory action, as evidenced by the inhibition percentage value.

The ability of honey to partially maintain its inhibitory effect on mature biofilms or even enhance its action in some instances is particularly significant. This is because mature biofilms undergo considerable alterations in bacterial cells, leading to increased virulence and resistance to conventional therapies [42,43]. The MTT test findings indicate that honey’s mechanisms of action are diverse, with its effect on metabolism not always being the dominant factor, suggesting that honey may employ multiple strategies to counteract biofilms and address the challenges.

The minimal or no inhibitory effects exhibited by almond and hawthorn honey on the biofilm formation of *P. aeruginosa* and *K. pneumoniae* suggest that the choice of honey may have valuable applications in controlling bacterial biofilms at the beginning of biofilm formation. This underlines the strain-specific nature of honey’s antibiofilm properties and its potential use in targeted applications.

### 3.2. Swimming Motility

Bacterial motility enables cells to spread across surfaces and move away from specific infection sites [44,45,46]. It can be essential for their colonization and biofilms’ subsequent formation. Swimming motility is also associated with the expression of virulent genes, the ability to invade human cells, and increased antibiotic resistance. The structures responsible for bacterial motility are intricate nanomachines composed of multiple components. Several essential mechanisms present promising targets to disrupt bacterial movement by interfering with chemotaxis and motility-related structures’ synthesis, assembly, and function. High-throughput screening of compounds that broadly inhibit motility or chemotaxis can be designed to assess the swimming behavior of specific pathogenic bacteria [46]. Our findings for Rosaceae honey corroborate the inhibitory effect of more known honey on bacterial motility and provide a fresh viewpoint within the existing research [14]. Manuka honey has been shown to suppress the swimming activity of *P. aeruginosa*, decreasing it from 75% to 62% when applied at a 12% (*w*/*v*) concentration [47]. Honey produced by the stingless bee *Melipona beecheii* affected the motility of *E. coli* and *S. aureus* [48]. The control of the motility of pathogens is, as known, another important mechanism to inhibit or limit their virulence. *A. baumannii*, although it is more known for its twitching motility [49], can, therefore, also spread rapidly on different semisolid and certain abiotic surfaces by swimming [50,51]. This property can help it in the adherence to surfaces and in the production of biofilm [52]. Motility allows *E. coli* and *S. aureus* to migrate to a new area, facilitating biofilm expansion [53]. *K. pneumoniae* is generally a non-motile bacterium. However, different *K. pneumoniae* isolates showed the capacity to swim [54]. Therefore, the swimming behavior of the clinical isolate of *Klebsiella* used in our experiments, beginning with its activity under standard growth conditions, should not be unexpected. To our knowledge, this is the first study that has highlighted the ability of some Rosaceae honey to negatively affect the motility of pathogenic bacteria. Only one work is reported in scientific literature concerning the action of Slovakian hawthorn honey against wound-associated bacteria *Proteus mirabilis* and *Enterobacter cloacae* [55].

### 3.3. Prebiotic Activity of Honey

In addition to their well-documented technological applications, as demonstrated, for instance, by Pannella et al. [56] and Colautti et al. [26], several lactic acid bacteria are also widely recognized for their probiotic properties, which contribute significantly to human health [34]. The beneficial effects of probiotic bacteria can be further enhanced by the presence of specific molecules, molecular classes, extracts, or functional ingredients in their environment, collectively referred to as prebiotics. Thus, while it has been well ascertained that honey can improve the gut microbial balance [12], the interplay between Rosaceae honey and probiotic lactobacilli is still underexplored. It could be hypothesized that the complex of its molecules can promote the growth of specific probiotic strains. Our findings align with previous studies demonstrating a growth-stimulating effect of honey on certain probiotic microorganisms. Chick et al. (2001), Rosendale et al. (2008), and Kajiwara et al. (2002) observed a positive effect on the biological properties of lactobacilli and bifidobacteria, such as the production of specific organic acids [57,58,59]. Monofloral honey can stimulate the growth and the activity of probiotics, too. Popa et al. demonstrated a positive impact of sourwood and *Medicago* on the growth of *L. bulgaricus* [60]. On the other hand, different types of *Eucalyptus* honey stimulated the growth of *L. plantarum* and *L. rhamnosus* [61], and lime honey can affect the cytotoxicity of lactobacilli grown in the presence of lime honey [62]. Shamala et al. (2000) demonstrated through in vitro and in vivo studies that the presence of honey, as a replacement for conventional sugar in the culture medium, could result in more than a tenfold increase in the growth of *L. plantarum*. Furthermore, the in vivo research revealed a significant rise in the number of viable lactic acid bacteria in the intestines of rats fed honey compared to those fed sucrose [63]. However, our results differ from those of Kgozeimeh et al. [64], who observed that honey had an inhibitory effect on the growth of *L. casei* and *L. rhamnosus;* therefore, our results do not align with those of Fratianni et al. [65], who reported that legume honey strongly promoted the growth of *L. gasseri* but without an evident effect on *L. rhamnosus*. Honey’s prebiotic activity, demonstrated in our experiments from the growth-stimulating effect and the hydrophobicity of probiotics, assumes particular importance, considering the role of probiotics in general in the host’s health. Rosaceae honey has, in some cases, demonstrated notable prebiotic properties [12,52], exerting a beneficial effect not only on the growth of lactic acid bacteria but also on their hydrophobicity—an important factor in assessing the potential of probiotics to adhere to intestinal epithelial cells [35,36]. The ability of supernatants from various lactic acid bacteria to exert antibacterial and antibiofilm effects has been extensively documented. More recently, this was confirmed by Pompilio et al. (2024) against certain *Pseudomonas aeruginosa* strains and by Saini et al. (2024), who demonstrated the inhibition of biofilm formation in ESKAPE pathogens by probiotic bacteria derived from the caprine gut [66,67]. The presence of honey can enhance the inhibitory potential of probiotics. As highlighted in previous studies, such as that by Nazzaro et al. [19], this effect may vary depending on the type of honey used as a fermentable substrate and the specific probiotic strain being analyzed. The antibiofilm activity of probiotic supernatants cultured with Rosaceae honey, particularly their ability to inhibit the metabolism of sessile pathogenic cells, could be highly significant given the overall positive impact of probiotics on host health. Thus, Rosaceae honey might influence pathogenic microorganisms directly while also playing an indirect role by enhancing the growth and activity of probiotics, which, in turn, produce biomolecules with inhibitory effects on harmful bacteria. These aspects also assume relevance in the case of the development of innovative probiotic-honey-based functional foods [68]. Future studies will also focus on the biochemical characterization of Rosaceae honey to better understand how their specific compositions influence their biological properties. The fermentation of the honey by probiotics certainly gave rise to metabolites classified as postbiotics [69], which certainly will deserve further future investigations from a biochemical identification point of view. Studies are ongoing to characterize such kinds of honey from a biochemical perspective, such as the qualitative and quantitative characterization of polyphenols and the study of volatile compounds. This aspect could provide interesting insights and lead to further studies on why one particular monofloral honey may exhibit greater inhibitory activity against pathogens compared to another. The biochemical composition and the response that different types of honey, such as Rosaceae honey, have against pathogens could also represent the basis for subsequent in vitro studies on other biological properties of honey, such as its anti-inflammatory effect and its inhibition of the key enzymes involved in neurodegenerative mechanisms, which, as we know, are often linked to the presence of some pathogens, including those used in this study, within the neurological system. These findings, previously ascertained for other kinds of honey [10,70,71,72,73], could also provide valuable insights into the potential of Rosaceae honey in combating neurodegenerative diseases, which are often associated with microbiome imbalances. The identification of a particular type of honey may also prove valuable in the field of microbiological nanotechnology. Honey is gaining significant attention in this area, particularly as part of the growing trend toward using eco-friendly ingredients. For instance, Tang et al. (2019) created an electrospun nanofibrous membrane made of alginate/PVA and infused with honey to develop an effective wound dressing material with enhanced antioxidant and antibacterial properties [74]; more recently, De La Mora et al. (2024) incorporated honey into PVA/chitosan/collagen nanofibrous membranes, resulting in nanofibers with strong antibacterial effects, excellent biocompatibility, and a growth-promoting influence on certain cell lines. This suggests their potential as antibacterial dressings for treating skin ulcers [75].

## 4. Materials and Methods

We used five commercial kinds of Rosaceae honey: hawthorn (“Biologique choice”, London, UK), cherry (“Thun”, Trentino, Italy), raspberry (“Biologique choice”, London, UK), almond (“Torrons I Mel Alemany”, Lleida, Spain), and apple (“Thun”, Italy). The companies highlighted the monofloral nature of the honey, which was produced under good agricultural practice, without the use of pesticides. Therefore, they could only be present in the Italian market in this way if they respected Italian law 179 of 2004. A total of 20 g of each kind of honey was energetically mixed with ultrapure water (1:4 *w*/*v*) [56] and filtered (0.45 µm; Millipore, Milano, Italy). The bacterial strains *Acinetobacter baumannii* (ATCC 19606), *Escherichia coli* (DSM 8579), *Pseudomonas aeruginosa* (DSM 50071), *Staphylococcus aureus* subsp. *aureus* Rosebach (ATCC 25923), *Listeria monocytogenes* (ATCC 7644), and a hospital isolate of *Klebsiella pneumoniae*, which was freshly cultured, as utilized in the experiments, were cultured in Luria Broth for 18 h at 37 °C or 35 °C and 80 rpm (Corning LSE, Pisa, Italy) (depending on the strain) before the experiments. Resazurin, crystal violet, MTT, and DMSO were purchased by Sigma Aldrich (Milan, Italy).

### 4.1. Minimal Inhibitory Concentration (MIC)

The minimal resazurin microtiter-plate assay was applied to evaluate the MIC [17,76] in flat-bottomed 96-well microtiter plates and then incubated at 37 °C for 24 h (*A. baumannii* grew at 35 °C under the same conditions). Sterile DMSO and tetracycline (dissolved in DMSO, 1 mg/mL, Sigma Aldrich) represented the negative and positive controls, respectively. The resazurin color change was determined in triplicate, and the results were expressed as the arithmetic mean ± standard deviation.

### 4.2. Antibiofilm Activity Exhibited by the Honey

The ability of honey to influence bacterial biofilm formation was evaluated using the crystal violet test (provided by Sigma Aldrich, Italy) on flat-bottomed 96-well microtiter plates (Falcon, VWR International, Milan, Italy) [77,78]. Ten microliters of overnight bacterial cultures (standardized to 0.5 McFarland using fresh culture broth) were added to each well along with 20 µg/mL of honey and sterile Luria–Bertani broth (LB, Sigma Aldrich Italy), reaching a final volume of 250 µL. The plates were sealed with parafilm tape and incubated for 48 h at 37 °C or 35 °C, depending on the bacterial strain. Following incubation, planktonic cells were removed, and sessile cells were gently rinsed twice with sterile phosphate-buffered saline (PBS). After 10 min, 200 µL of methanol was added to fix the sessile cells for 15 min, after which it was discarded. Once the plates had dried, 200 µL of 2% *w*/*v* crystal violet solution was added to each well for 20 min. The staining solution was removed, and the wells were washed gently with sterile PBS and left to dry. Finally, 200 µL of 20% *w*/*v* glacial acetic acid was added to release the bound dye, and absorbance was measured at 540 nm (Cary 50 Bio, Varian, Palo Alto, CA, USA). The adhesion percentage was calculated relative to a control (bacteria grown without honey, with no inhibition assumed). All experiments were conducted in triplicate, and results were expressed as mean ± SD. For mature biofilm, 10 µL of overnight bacterial cultures (standardized to 0.5 McFarland using Luria–Bertani broth) were added to 96-well flat-bottomed plates to reach a final volume of 250 µL per well. The plates were sealed with parafilm tape to prevent evaporation and incubated at 37 °C (35 °C for *A. baumannii*) for 24 h. After planktonic cells were removed, honey was added at a concentration of 20 µg/mL, and Luria–Bertani broth was added to have a final volume of 250 µL. Plates were then incubated for another 24 h. The experimental steps, including inhibition percentage calculation relative to untreated bacteria, were performed as described previously.

### 4.3. Evaluation of Honey’s Effect on the Metabolic Activity of Sessile Cells

The impact of honey (20 µg/mL) on bacterial metabolic activity was analyzed by adding it either at the start of bacterial growth or after 24 h. This assessment utilized the (3-(4,5-Dimethylthiazol-2-yl)-2,5-Diphenyltetrazolium Bromide, Sigma Aldrich, Italy) MTT colorimetric assay [77,78]. After 48 h of total incubation, planktonic cells were removed, and 150 µL of PBS and 30 µL of 0.3% MTT (Sigma, Milan, Italy) were added to each well. Plates were incubated for 2 h at 37 °C or 35 °C, depending on the bacterial strain. Afterward, the MTT solution was discarded, and wells were washed twice with 200 µL of sterile PBS. Formazan crystals were dissolved by adding 200 µL of DMSO, and absorbance was measured at 570 nm (Cary 50 Bio Varian).

### 4.4. Swimming Motility of Pathogens

In total, 4 μL of bacteria from overnight-grown colonies were stabbed on the surface of the 0.4% semisolid Luria–Bertani medium. The agar plates were then incubated at 37 °C for 48 h. Swimming motility was measured with the distance of bacterial growth away from the inoculation point using a sliding caliper. The measurement of each sample was replicated three times in the same Petri dishes.

### 4.5. Prebiotic Activity of the Honey

*Lacticaseibacillus casei* Shirota (LcS), *Lactobacillus gasseri* LG050, *Lactiplantibacillus plantarum* 299 V, and *Lacticaseibacillus rhamnosus* GG were obtained from commercial formulation available in a local pharmacy. *Lactobacillus bulgaricus* (DSM 20081) was provided by DMSZ (Braunschweig-Süd, Germany).

#### 4.5.1. Growth of Lactic Acid Bacteria in the Presence of Honey

The strains were grown at 37 °C (except *L. plantarum*, which was grown at 30 °C) for 16–18 h in MRS without glucose (Liofilchem, Roseto degli Abruzzi, Italy), which was substituted by an equal concentration (*weight*/*vol*) of the five honeys. The growth was read at λ = 600 nm (Cary 50Bio, Varian, Palo Alto, CA, USA). The effect of the five honeys on the growth of the lactic bacteria was calculated as a percentage compared to the control when the strains were grown in the presence of glucose.

#### 4.5.2. Microbial Adhesion to Solvent

The microbial adhesion to solvent (MAS) test was performed according to Nazzaro et al. [19]. First, LAB cells were washed with sterilized isotonic saline (0.9%), harvested, and re-suspended in the same solution so that the final concentration of intact cells was the same as in the initial experiment. The absorbance of the cell suspension (A0) was measured at λ = 600 nm (Cary50Bio Varian); then, we added an equal volume of xylene and mixed the two-phase system thoroughly by continuous vortexing for 3 min. The aqueous phase was removed after one hour of incubation at 37 or 30 °C (depending on the strain), and its absorbance (A1) was measured. The adhesion was calculated from three replicates as a percentage decrease in the optical density of the original bacterial suspension using the following formula: % = [(A0 − A1)/A0] · 100.

#### 4.5.3. Antibiofilm Activity of the Supernatants of the LAB Grown in the Presence of the Honey

The five LAB strains were grown at 37 °C for 18 h in an MRS medium, where glucose was substituted by an equal concentration (*w*/*v*) of the honey. Following centrifugation (3000 rpm, 4 °C, 10 min), the supernatant was recovered, filtered (mesh 0.22 μm, Merck Life Science, Milano, Italy), and maintained at −20 °C until the antibiofilm tests.

For the biofilm inhibition test, the pathogenic strains were grown in Luria–Bertani broth at 37 °C (*A. baumannii* was incubated at 35 °C) for 18 h [17]. Ten microliters of each bacterial culture were added to a multi-well plate, previously filled with 80 μL/mL of LAB culture supernatant and Luria–Bertani (Merck Life Science, Milano, Italy) broth up to a final volume of 250 μL. After 24 h of incubation, the inhibitory effect on the adhesion process of pathogens was evaluated using the previously described crystal violet and MTT test, measuring it as the percent with respect to the control (untreated pathogenic bacteria) for which an inhibition = 0% was assumed.

### 4.6. Statistical Analysis

Results were expressed as the mean ± SD of three experiments (PC software “Excel Statistics” version 365). One-way analysis of variance (ANOVA) with a high confidence level (95%, *p* < 0.05) was applied to evaluate and compare the differences between samples. Student’s *t*-tests were used to examine the mean values of the results.

## Figures and Tables

**Table 1:** Minimal Inhibitory Concentration of honey (μg/mL). Results are the average (±SD) of three independent experiments. *AB = A. baumannii*; *EC = E. coli*; *KP* =*K. pneumoniae*; *LM* = *L. monocytogenes*; *PA = P. aeruginosa*; *SA = S. aureus*. Honey: hawthorn (H); cherry (CH); raspberry (R); almond (AL); apple (AP). As control (C), we used tetracycline. ^a^: *p* < 0.5; ^b^: *p* < 0.01 (ANOVA followed by Dunnett’s multiple comparison test).

MIC	*AB*	*EC*	*KP*	*LM*	*PA*	*SA*
H	34.0 ± 2.0 ^a^	34.0 ± 1.0 ^a^	>50 ^b^	33.0 ± 1.0 ^a^	34.0 ± 1.0 ^a^	32.0 ± 1.0 ^a^
CH	32.0 ± 1.0 ^a^	34.0± 1.0 ^a^	40.0 ± 2.0 ^a^	32.0 ± 1.0 ^a^	>50 ^b^	32.0 ± 1.0 ^a^
R	32.0 ± 1.0 ^a^	32.0± 1.0 ^a^	>50 ^b^	32.0± 1.0 ^a^	40.0± 2.0 ^a^	40.0 ± 2.0 ^a^
AL	32.0 ± 1.0 ^a^	32.0± 1.0 ^a^	>50 ^b^	33.0± 1.0 ^a^	>50 ^b^	40.0 ± 2.0 ^a^
AP	33.0 ± 2.0 ^a^	30.0± 1.0 ^a^	>50 ^b^	34.0± 1.0 ^a^	40.0 ± 2.0 ^a^	40.0 ± 1.0 ^a^
C	28.0 ± 1.0	28.0 ± 2.0	32.0± 1.0 ^a^	30.0 ± 2.0	28.0 ± 2.0	34.0 ± 1.0

**Table 2:** Biofilm inhibitory activity (expressed as percentages) of the five types of Rosaceae honey, added at zero (CV0) and after 24 h (CV24) against the pathogens *A. baumannii* (*AB*), *E. coli* (*EC*), *K. pneumoniae* (*KP*), *L. monocytogenes* (*LM*), *P. aeruginosa* (*PA*), and *S. aureus* (*SA*), and assuming for the control (untreated bacteria) an inhibitory value = 0. Honey: hawthorn (H); cherry (CH); raspberry (R); almond (AL); apple (AP). Results are reported as the mean ± SD of three experiments; ^a^: *p* < 0.05 and ^b^: *p* < 0.01, ^c^: *p* < 0.001 according to two-way ANOVA.

CV 0 (%)	*AB*	*EC*	*KP*	*LM*	*PA*	*SA*
H	57.94 ± 0.37 ^c^	47.82 ± 0.27 ^c^	6.89 ± 0.91 ^a^	55.87 ± 0.18 ^c^	4.24 ± 0.99 ^a^	46.25 ± 0.26 ^c^
CH	43.49 ± 0.11 ^b^	55.39 ± 0.67 ^c^	29.66 ± 0.87 ^b^	54.89 ± 0.35 ^c^	0.00 ± 0.00	51.25 ± 0.35 ^c^
R	54.60 ± 0.71 ^c^	52.99 ± 0.54 ^c^	0.00 ± 0.00	54.40 ± 0.55 ^c^	31.88 ± 0.8 ^b^	34.30 ± 0.38 ^b^
AL	52.24 ± 0.28 ^c^	57.59 ± 0.27 ^c^	0.00 ± 0.00	48.08 ± 0.30 ^c^	7.86 ± 0.14 ^a^	28.76 ± 0.45 ^b^
AP	59.43 ± 0.21 ^c^	54.16 ± 0.57 ^c^	0.00 ± 0.00	44.53 ± 0.45 ^b^	22.73 ± 0.12 ^b^	39.86 ± 0.67 ^b^
CV24 (%)	*AB*	*EC*	*KP*	*LM*	*PA*	*SA*
H	5.05 ± 0.08 ^a^	0.00 ± 0.00	0.00 ± 0.00	0.00 ± 0.00	26.26 ± 0.15 ^b^	0.00 ± 0.00
CH	2.29 ± 0.20 ^a^	0.00 ± 0.00	0.00 ± 0.00	0.00 ± 0.00	20.23 ± 0.16 ^a^	39.95 ± 0. 05 ^b^
R	14.33 ± 0.35 ^a^	0.00 ± 0.00	1.17 ± 0.04	0.00 ± 0.00	12.48 ± 0.41 ^a^	30.13 ± 0.15 ^b^
AL	12.87 ± 0.13 ^a^	0.00 ± 0.00	0.00 ± 0.00	33.43 ± 0.26 ^b^	33.08 ± 0.19 ^b^	10.95 ± 0.11 ^a^
AP	9.71 ± 0.54 ^a^	0.77 ± 0.06	21.58 ± 0.94 ^a^	18.82 ± 0.13 ^a^	38.01 ± 0.94 ^b^	20.11 ± 0.15 ^a^

**Table 3:** Inhibitory activity (expressed as percentages) of the five types of Rosaceae honey, added at time zero (MTT0) and after 24 h of incubation (MTT24) against the metabolism of sessile cells of pathogens *A. baumannii* (*AB*), *E. coli* (*EC*), *K. pneumoniae* (*KP*), *L. monocytogenes* (*LM*), *P. aeruginosa* (*PA*), and *S. aureus* (*SA*), assuming for the control (untreated bacteria) an inhibitory value = 0. Honey: hawthorn (H); cherry (CH); raspberry (R); almond (AL); apple (AP). Results are reported as the mean ± SD of three experiments, ^a^: *p* < 0.05, ^b^ *p* < 0.01, ^c^: *p* < 0.001, according to two-way ANOVA.

MTT 0 (%)	*AB*	*EC*	*KP*	*LM*	*PA*	*SA*
H	0.00 ± 0.00	27.38 ± 0.84 ^b^	52.66 ± 0.32 ^c^	50.16 ± 2.01 ^c^	19.83 ± 1.13 ^a^	0.00 ± 0.00
CH	27.16 ± 0.04 ^b^	32.96 ± 0.40 ^b^	56.47 ± 0.56 ^c^	51.32 ± 1.45 ^c^	25.66 ± 0.32 ^b^	0.00 ± 0.00
R	0.00 ± 0.00	35.98 ± 0.62 ^b^	43.78 ± 1.67 ^b^	53.39 ± 0.43 ^c^	12.86 ± 0.21 ^a^	0.00 ± 0.00
AL	2.57 ± 0.09 ^a^	30.18 ± 0.51 ^b^	33.08 ± 0.57 ^b^	46.90 ± 0.56 ^c^	1.78 ± 0.87 ^a^	0.00 ± 0.00
AP	0.00 ± 0.00	25.37 ± 0.32 ^b^	24.24 ± 0.26 ^b^	40.27 ± 0.38 ^b^	0.23 ± 0.21	0.00 ± 0.00
MTT 24 (%)	*AB*	*EC*	*KP*	*LM*	*PA*	*SA*
H	37.57 ± 2.45 ^b^	0.00 ± 0.00	0.00 ± 0.00	0.00 ± 0.00	0.00 ± 0.00	0.00 ± 0.00
CH	0.00 ± 0.00	0.00 ± 0.00	0.00 ± 0.00	5.36 ± 0.31 ^a^	0.00 ± 0.00	0.00 ± 0.00
R	32.20 ± 2.64 ^b^	0.00 ± 0.00	0.00 ± 0.00	0.00 ± 0.00	15.91 ± 0.62 ^a^	0.00 ± 0.00
AL	46.47 ± 3.76 ^c^	0.00 ± 0.00	0.00 ± 0.00	1.70 ± 0.16 ^a^	0.00 ± 0.00	5.17 ± 0.33 ^a^
AP	54.36 ± 3.67 ^c^	41.54 ± 2.23 ^b^	0.00 ± 0.00	5.49 ± 0.11 ^a^	0.00 ± 0.00	19.52 ± 1.57 ^a^

**Table 4:** Swimming of pathogenic bacteria grown with the presence of hawthorn (H), cherry (CH), raspberry (R), almond (AL), and apple (AP) Rosaceae honey, or in the presence of glucose, which was the control (CO). Results are expressed in mm and represent three independent experiments’ averages (DS). *A. baumannii* (*AB*), *E. coli* (*EC*), *K. pneumoniae* (*KP*), *L. monocytogenes* (*LM*), *P. aeruginosa* (*PA*), and *S. aureus* (*SA*). Results are reported as the mean±SD of three experiments; ^a^: *p* < 0.05, ^b^ *p* < 0.01, and ^c^: *p* < 0.001 according to two-way ANOVA.

	*AB*	*EC*	*KP*	*LM*	*PA*	*SA*
H	10 ±1.0 ^c^	10 ± 1.5 ^a^	12 ± 0.5 ^a^	10 ±1.0 ^a^	8 ± 0.5 ^a^	15 ± 0.5
CH	15 ±1.0 ^b^	7 ± 0.5 ^b^	10 ±1.0 ^a^	7 ±1.0 ^a^	7 ±1.0 ^a^	15 ± 1.5
R	15 ±1.5 ^b^	15± 1.5	12 ± 1.5 ^a^	7 ±1.0 ^a^	13 ±2.0 ^a^	10 ±1.5 ^a^
AL	18 ± 1.5 ^b^	15± 1.5	15 ± 1.0 ^a^	10 ±2.0 ^a^	9 ±1.0 ^a^	10 ± 0.6 ^a^
AP	20 ± 1.0 ^a^	10 ±1.0 ^a^	13 ± 0.5 ^a^	10 ± 1.5 ^a^	13 ±1.0 ^a^	10 ± 0.5 ^a^
CO	25 ± 1.0	15 ± 1.5	15 ± 1.5	12 ± 1.0	12 ± 1.5	15 ± 1.0

**Table 5:** Growth of probiotics with or without (control) the presence of Rosaceae honey. The data are expressed in terms of λ = 600 nm and are reported as the average (±SD) of three independent experiments. Honey: hawthorn (H); cherry (CH); raspberry (R); almond (AL); apple (AP). CO represents the growth in conventional MRS containing glucose. *LB*: *L. bulgaricus*; *LC*: *L. casei* Shirota; *LG*: *L. gasseri*; *LP*: *L. plantarum*; *LR*: *L. rhamnosus*. Results are reported as the mean ± SD of three experiments; ^b^ *p* < 0.01, and ^d^: *p* < 0.0001 according to two-way ANOVA.

	CO	H	CH	R	AL	AP
*LB*	0.603 ± 0.17	1.348 ± 0.11 ^b^	1.222 ± 0.11 ^b^	1.302 ± 0.12 ^b^	1.332 ± 0.14 ^b^	1.392 ± 0.15 ^b^
*LC*	0.504 ± 0.11	1.512 ± 0.12 ^b^	1.323 ± 0.14 ^b^	1.622 ± 0.14 ^b^	1.470 ± 0.15 ^b^	1.483 ± 0.14 ^b^
*LG*	0.449 ± 0.08	1.377 ± 0.13 ^b^	1.296 ± 0.11 ^b^	1.431 ± 0.14 ^b^	1.387 ± 0.14 ^b^	1.385 ± 0.11 ^b^
*LP*	0.125 ± 0.07	1.431 ± 0.13 ^d^	1.331 ± 0.12 ^d^	1.351 ± 0.11 ^d^	1.403 ± 0.13 ^d^	1.442 ± 0.13 ^d^
*LR*	0.182 ± 0.06	1.412 ± 0.13 ^d^	1.376 ± 0.14 ^d^	1.435 ± 0.13 ^d^	1.441 ± 0.14 ^d^	1.445 ± 0.14 ^d^

**Table 6:** Hydrophobicity of probiotics evaluated after 3 h of contact with the organic solvent xylene. The data are expressed in terms of % and are reported as the average (±SD) of three independent experiments. Honey: hawthorn (H); cherry (CH); raspberry (R); almond (AL); apple (AP). CO represents the growth in conventional MRS containing glucose—*LB*: *L. bulgaricus*; *LC*: *L. casei* Shirota; *LG*: *L. gasseri*; *LP*: *L. plantarum*; *LR*: *L. rhamnosus*. Results are reported as the mean ± SD of three experiments: ^a^: *p* < 0.05 according to two-way ANOVA.

	CO	H	CH	R	AL	AP
*LB*	3.71 ± 0.25	10.5 ± 1.13 ^a^	4.66 ± 0.54	8.95 ± 0.77 ^a^	12.7 ± 1.15 ^a^	7.97 ± 0.67 ^a^
*LC*	0.50 ± 0.00	4.23 ± 0.31 ^a^	0.47 ± 0.00	10.13 ± 1.21 ^a^	2.26 ± 0.14 ^a^	0.84 ± 0.71
*LG*	3.12 ± 0.28	0.12 + 0.02	0.10 + 0.02	6.57 ± 0.53 ^a^	0.09 ± 0.01	4.07 ± 0.35 ^a^
*LP*	0.78 ± 0.00	0.30 + 0.03	5.87 ± 0.60 ^a^	0.36 + 0.02	0.32 ± 0.02	0.34 + 0.02
*LR*	0.89 ± 0.09	0.72 + 0.06	0.85 + 0.08	0.74 + 0.09	0.76 ± 0.09	0.80 + 0.06

**Table 7:** Inhibitory activity of *L. bulgaricus*, *L. casei* Shirota, *L. gasseri*, *L. plantarum*, and *L. rhamnosus* supernatant against the biofilm of *A. baumannii* (*AB*), *E. coli* (*EC*), *K. pneumoniae* (*KP*), *L. monocytogenes* (*LM*), *P. aeruginosa* (*PA*), and *S. aureus* (*SA*). The probiotic strain was grown with or without the presence of honey. Honey—hawthorn (H), cherry (CH), raspberry (R), almond (AL), and apple (AP)—was used as substitutes for glucose. CO represents the growth in conventional MRS containing glucose. Results are reported as the mean±SD of three experiments; ^a^: *p* < 0.05, ^b^ *p* < 0.01, and ^c^: *p* < 0.001 according to two-way ANOVA.

CV		H	CH	R	AL	AP	CO
*L. bulgaricus*	*AB*	47.68 ± 3.33 ^b^	49.33 ± 4.04	42.94 ± 4.04 ^b^	26.71 ± 1.57 ^a^	38.14 ± 2.22 ^b^	45.76 ± 3.57 ^b^
	*EC*	0.00 ± 0.00	0.00 ± 0.00	0.00 ± 0.00	8.43 ± 0.57 ^a^	0.00 ± 0.00	0.91 ± 0.57
	*KP*	72.15 ± 4.44 ^c^	0.00 ± 0.00	0.00 ± 0.00	0.00 ± 0.00	0.00 ± 0.00	33.06 ± 2.34 ^b^
	*LM*	44.35 ± 4.01 ^b^	0.00 ± 0.00	8.90 ± 1.02 ^a^	11.92 ± 0.78 ^a^	15.20 ± 0.67 ^a^	17.69 ± 1.33 ^a^
	*PA*	29.28 ± 2.03 ^b^	0.00 ± 0.00	29.62 ± 3.01 ^b^	4.03 ± 0.57 ^a^	13.47 ± 1.02 ^a^	0.00 ± 0.00
	*SA*	10.05 0.56 ^a^	18.07 ± 0.98 ^a^	19.80 ± 1.45 ^a^	5.87 ± 1.02 ^a^	26.58 ± 2.04 ^b^	5.07 ± 0.13 ^a^
*L. casei*	*AB*	44.97 ± 3.67 ^b^	44.77 ± 4.03 ^b^	38.24 ± 3.01 ^b^	57.99 ± 2.25 ^c^	53.71 ± 4.01 ^c^	43.01 ± 3.98 ^b^
	*EC*	0.00 ± 0.00	0.00 ± 0.00	9.59 ± 1.02 ^a^	0.00 ± 0.00	26.35 ± 2.04 ^b^	18.96 ± 1.03 ^a^
	*KP*	72.15 ± 2.34 ^c^	0.00 ± 0.00	0.00 ± 0.00	0.00 ± 0.00	30.62 ± 2.2 ^b^	34.22 ± 2.87 ^b^
	*LM*	44.35 ± 3.34 ^b^	0.00 ± 0.00	19.46 ^a^ ± 1.03	30.31 ± 2.92 ^b^	21.35 ± 1.44 ^a^	63.95 ± 1.25 ^c^
	*PA*	29.28 ± 1.55 ^b^	0.00 ± 0.00	0.00 ± 0.00	0.00 ± 0.00	0.00 ± 0.00	20.82 ± 2.03 ^a^
	*SA*	10.05 ± 0.67 ^a^	18.07 ± 1.13 ^a^	0.00 ± 0.00	0.00 ± 0.00	0.00 ± 0.00	25.80 ± 2.09 ^b^
*L. gasseri*	*AB*	14.63 ± 1.02 ^a^	23.83 ± 1.44 ^a^	25.75 ± 2.01 ^b^	12.31 ± 0.2 ^a^	14.23 ± 1.02 ^a^	29.45 ± 2.09 ^b^
	*EC*	0.00 ± 0.00 ^b^	0.00 ± 0.00 ^b^	0.00 ± 0.00 ^b^	0.00 ± 0.00 ^b^	0.00 ± 0.00 ^b^	21.13 ± 1.17
	*KP*	0.00 ± 0.00	0.00 ± 0.00	0.00 ± 0.00	0.00 ± 0.00	0.00 ± 0.00	0.00 ± 0.00
	*LM*	0.00 ± 0.00	0.00 ± 0.00	0.00 ± 0.00	0.00 ± 0.00	0.00 ± 0.00	0.00 ± 0.00
	*PA*	0.00 ± 0.00	34.43 ± 1.57 ^b^	0.00 ± 0.00	0.00 ± 0.00	0.00 ± 0.00	0.00 ± 0.00
	*SA*	7.02 ± 1.90 ^a^	60.62 ± 2.44 ^c^	28.02 ± 2.12 ^b^	0.00 ± 0.00	27.72 ± 2.09 ^b^	0.00 ± 0.00
*L. plantarum*	*AB*	50.71 ± 4.03 ^c^	13.27 ± 0.57 ^a^	42.02 ± 2.32 ^b^	39.68 ± 3.01 ^b^	24.05 ± 2.12 ^a^	0.00 ± 0.00
	*EC*	0.00 ± 0.00	0.00 ± 0.00	0.00 ± 0.00	14.51 ^a^ ± 1.02	7.53 ^a^ ± 0.57	22.23 ^a^ ± 0.67
	*KP*	0.00 ± 0.00	1.44 ± 0.05	0.00 ± 0.00	1.92 ± 0.06	0.00 ± 0.00	0.00 ± 0.00
	*LM*	0.00 ± 0.00	0.00 ± 0.00	0.00 ± 0.00	0.00 ± 0.00	0.00 ± 0.00	0.00 ± 0.00
	*PA*	0.00 ± 0.00	22.97 ± 0.23 ^a^	0.00 ± 0.00	24.69 ± 1.34 ^a^	26.52 ± 1.55 ^b^	37.63 ± 1.67 ^b^
	*SA*	24.93 ± 2.01 ^a^	21.90 ± 0.57 ^a^	0.00 ± 0.00	15.48 ± 0.13 ^a^	27.01 ± 1.71 ^b^	38.59 ± 2.04 ^b^
*L. rhamnosus*	*AB*	0.00 ± 0.00	0.00 ± 0.00	0.00 ± 0.00	0.00 ± 0.00	0.00 ± 0.00	0.00 ± 0.00
	*EC*	0.00 ± 0.00	0.00 ± 0.00	0.00 ± 0.00	0.00 ± 0.00	0.00 ± 0.00	2.95 ± 0.07 ^a^
	*KP*	3.12 ± 0.05 ^a^	13.06 ± 0.56 ^a^	0.00 ± 0.00	0.00 ± 0.00	0.00 ± 0.00	6.81 ± 0.11 ^a^
	*LM*	0.00 ± 0.00	0.00 ± 0.00	0.00 ± 0.00	0.00 ± 0.00	0.00 ± 0.00	0.00 ± 0.00
	*PA*	44.16 ± 1.98 ^c^	14.91 ± 1.02 ^a^	0.47 ± 0.03	0.00 ± 0.00	9.28 ± 0.77 ^a^	0.00 ± 0.00
	*SA*	16.99 ± 0.32 ^a^	0.00 ± 0.00	0.00 ± 0.00	0.00 ± 0.00	0.00 ± 0.00	0.00 ± 0.00

**Table 8:** Inhibitory activity exhibited by the culture supernatants of probiotics *L. bulgaricus*, *L. casei* Shirota, *L. gasseri*, *L. plantarum*, and *L. rhamnosus* grown in the presence of hawthorn (H), cherry (CH), raspberry (R), almond (AL), and apple (AP) honey, or in the conventional MRS (CO) versus the metabolism of sessile cells of *A. baumannii* (AB), *E. coli* (EC), *K. pneumoniae* (KP), *L. monocytogenes* (LM), *P. aeruginosa* (PA), and *S. aureus* (SA). Data are reported as a percentage of inhibition and calculated with respect to the untreated pathogenic bacteria, for whom the inhibition was assumed to be zero. The results reported are the mean ± SD of three experiments, ^a^: *p* < 0.05, ^b^ *p* < 0.01, ^c^: *p* < 0.001, and ^d^: *p* < 0.0001, according to two-way ANOVA.

MTT		H	CH	R	AL	AP	MRS
*L. bulgaricus*	*AB*	11.83 ± 0.57 ^a^	41.14 ± 1.75 ^b^	55.13 ± 5.01 ^c^	67.64 ± 4.43 ^c^	62.81 ± 4.07 ^c^	57.93 ± 4.41 ^c^
	*EC*	94.43 ± 1.66 ^d^	47.70 ± 2.22 ^b^	35.94 ± 4.75 ^b^	44.39 ± 4.98 ^b^	43.54 ± 2.02 ^b^	34.04 ± 2.57 ^b^
	*KP*	0.00 ± 0.00	18.51 ± 1.76 ^a^	36.21 ± 1.87 ^b^	48.08 ± 1.34 ^b^	46.70 ± 1.13 ^b^	21.08 ± 0.67 ^a^
	*LM*	0.00 ± 0.00	0.00 ± 0.00	9.60 ± 0.00 ^a^	0.00 ± 0.00	14.09 ± 0.98 ^a^	10.95 ± 0.57 ^a^
	*PA*	0.00 ± 0.00	0.00 ± 0.00	0.81 ± 0.03 ^a^	0.00 ± 0.00	4.28 ± 0.10 ^a^	3.73 ± 0.11 ^a^
	*SA*	24.60 ± 1.21 ^a^	36.13 ± 2.31 ^b^	47.39 ± 3.81 ^b^	45.98 ± 3.07 ^b^	47.74 ± 4.44 ^b^	31.76 ± 3.11 ^b^
*L. casei*	*AB*	67.91 ± 4.57 ^c^	66.67 ± 3.76 ^c^	70.76 ± 2.12 ^c^	59.02 ± 3.87 ^c^	69.35 ± 3.76 ^c^	55.89 ± 4.21 ^c^
	*EC*	44.18 ± 1.57 ^b^	53.99 ± 3.02 ^c^	46.54 ± 2.98 ^b^	51.06 ± 3.02 ^b^	41.26 ± 3.15 ^b^	34.86 ± 2.78 ^b^
	*KP*	65.50 ± 2.43 ^b^	48.50 ± 3.26 ^b^	45.27 ± 3.21 ^b^	45.30 ± 3.88 ^b^	52.80 ± 3.01 ^b^	24.32 ± 0.57 ^a^
	*LM*	7.76 ± 1.11 ^a^	18.67 ± 2.44 ^a^	0.00 ± 0.00	0.00 ± 0.00	0.00 ± 0.00	12.70 ± 0.76 ^a^
	*PA*	26.30 ± 2.09 ^b^	19.46 ± 2.09 ^a^	2.62 ± 0.43 ^a^	0.00 ± 0.00	0.00 ± 0.00	0.00 ± 0.00
	*SA*	45.57 ± 3.54 ^b^	51.68 ± 2.98 ^b^	31.04 ± 1.67 ^b^	29.80 ± 1.12 ^b^	37.70 ± 1.45 ^b^	41.99 ± 1.24 ^b^
*L. gasseri*	*AB*	51.69 ± 4.01 ^b^	57.34 ± 4.87 ^c^	66.33 ± 3.01 ^c^	66.87 ± 2.44 ^c^	66.69 ± 2.57 ^c^	62.97 ± 3.01 ^c^
	*EC*	67.81 ± 3.91 ^c^	60.79 ± 3.65 ^c^	66.26 ± 2.87 ^c^	63.36 ± 2.09 ^c^	68.74 ± 2.76 ^c^	57.89 ± 1.67 ^c^
	*KP*	81.77 ± 1.45 ^d^	88.03 ± 1.98 ^d^	84.06 ± 1.57 ^d^	82.59 ± 1.13 ^d^	87.61 ± 1.13 ^d^	89.73 ± 1.43 ^d^
	*LM*	0.00 ± 0.00	0.00 ± 0.00	0.00 ± 0.00	0.00 ± 0.00	0.00 ± 0.00	0.00 ± 0.00
	*PA*	0.00 ± 0.00	0.00 ± 0.00	16.01 ± 0.14 ^a^	22.22 ± 0.12 ^a^	0.00 ± 0.00	1.90 ± 0.05 ^a^
	*SA*	0.00 ± 0.00	2.03 ± 0.06 ^a^	0.00 ± 0.00	19.31 ± 1.12 ^a^	22.42 ± 1.44 ^a^	26.11 ± 0.43 ^b^
*L. plantarum*	*AB*	36.79 ± 3.37 ^b^	32.14 ± 2.42 ^b^	58.47 ± 4.91 ^c^	34.52 ± 2.32 ^b^	40.52 ± 3.01 ^b^	0.00 ± 0.00
	*EC*	42.54 ± 1.11 ^b^	40.23 ± 2.02 ^b^	46.46 ± 3.98 ^b^	50.06 ± 3.34 ^b^	49.30 ± 3.01 ^b^	13.79 ± 2.67 ^a^
	*KP*	50.28 ± 1.57 ^b^	60.04 ± 2.67 ^c^	46.61 ± 2.25 ^b^	59.32 ± 3.91 ^c^	58.44 ± 4.67 ^c^	29.40 ± 1.57 ^b^
	*LM*	0.00 ± 0.00	0.00 ± 0.00	4.31 ± 0.34 ^a^	23.12 ± 0.57 ^a^	6.06 ± 0.43 ^a^	3.15 ± 0.05 ^a^
	*PA*	0.00 ± 0.00	16.30 ± 0.57 ^a^	0.00 ± 0.00	0.00 ± 0.00	13.45 ± 1.12 ^a^	0.00 ± 0.00
	*SA*	29.80 ± 2.91 ^b^	39.86 ± 3.05 ^b^	28.13 ± 2.03 ^b^	22.00 ± 1.13 ^a^	26.99 ± 1.67 ^b^	0.00 ± 0.00
*L. rhamnosus*	*AB*	16.08 ± 0.67 ^a^	36.52 ± 1.25 ^b^	41.27 ± 2.71 ^b^	46.06 ± 1.61 ^b^	35.21 ± 1.23 ^b^	14.22 ± 1.21 ^a^
	*EC*	53.09 ± 3.11 ^c^	33.41 ± 2.54 ^b^	52.95 ± 3.65 ^c^	52.55 ± 3.97 ^c^	56.43 ± 4.09 ^c^	59.68 ± 3.92 ^c^
	*KP*	53.81 ± 3.67	37.01 ± 0.89 ^b^	69.21 ± 2.13 ^c^	59.54 ± 3.03 ^c^	59.53 ± 4.12 ^c^	57.72 ± 4.24 ^c^
	*LM*	56.66 ± 2.09 ^c^	41.29 ± 4.65 ^b^	48.94 ± 3.74 ^b^	50.90 ± 4.45 ^b^	49.70 ± 4.21 ^b^	60.75 ± 3.12 ^c^
	*PA*	56.85 ± 1.14 ^c^	44.29 ± 3.85 ^b^	47.16 ± 3.05 ^b^	39.54 ± 4.01 ^b^	40.29 ± 1.43 ^b^	43.04 ± 3.41 ^b^
	*SA*	50.25 ± 5.21 ^c^	40.54 ± 3.67 ^b^	43.39 ± 3.13 ^b^	44.79 ± 4.35 ^b^	36.95 ± 3.02 ^b^	51.44 ± 4.15 ^c^

## Data Availability

The data presented in this study are available upon reasonable request.
